# The Role of ^18^F-FDG PET/CT in the Management of the Autoimmune Thyroid Diseases

**DOI:** 10.3389/fendo.2019.00208

**Published:** 2019-04-05

**Authors:** Bogdan Małkowski, Zbigniew Serafin, Rafał Glonek, Szymon Suwała, Rita Łopatto, Roman Junik

**Affiliations:** ^1^Department of Nuclear Medicine, Oncology Centre, prof. Łukaszczyk Memorial Hospital, Bydgoszcz, Poland; ^2^Department of Radiology and Diagnostic Imaging, University of Nicolaus Copernicus in Torun, Collegium Medicum in Bydgoszcz, Bydgoszcz, Poland; ^3^Department of Endocrinology and Diabetology, University of Nicolaus Copernicus in Torun, Collegium Medicum in Bydgoszcz, Bydgoszcz, Poland

**Keywords:** thyroid, ultrasound, autoimmune thyroid disease, 18FDG-PET, Hashimoto's disease

## Abstract

**Objective:** It is a well-known fact that positron emission tomography (PET) is an effective tool in the assessment of thyroid focal lesions, however only few studies so far have investigated its role in monitoring of autoimmune thyroid diseases (AITDs). The aim of this study is to assess if PET scan may be useful for the assessment of the thyroid gland in patients with an AITD—Hashimoto's thyroiditis.

**Methods:** We evaluated twenty subjects with diagnosed Hashimoto's thyroiditis (proven by presence of elevated thyroid antibodies level and by thyroid imaging). The maximum standardized uptake value (SUV-max) of the thyroid parenchyma was measured using ^18^F-FDG-PET/CT. Control group consisted of patients who have been in carcinoma remission for other reasons than thyroid cancer and who had been investigated by PET scan to exclude carcinoma recurrence.

All control group subjects had their thyroid glands intact, none of them had a medical history of thyroid disease including thyroid nodules. AITDs had been excluded in all control group subjects. STATISTICA 13.1 software was used for statistical analysis.

**Results:** Results: The SUV-max was significantly higher in patients with an AITD than in healthy subjects (median SUV-max 3.94 vs. 1.95; *p* = 0.005).

**Conclusions:** ^18^F-FDG-PET/CT scan may differentiate normal thyroid parenchyma from the diffused inflammatory changes of the thyroid gland in patients with AITDs. However, the researchers must be continued.

## Introduction

Autoimmune thyroid diseases (AITDs) is a wide group of autoimmune thyroid disorders like hyperthyroid Graves' disease, Hashimoto's thyroiditis or atrophic autoimmune hypothyroidism (1). The most common type of an AITD in Poland is Hashimoto's thyroiditis (referred to as AITD, throughout this article), which is much more common in women. Its prevalence is estimated at 2% in all age groups with an annual incidence of 0.3–1.5 per thousand people, however, a statistical growth of its incidence has been recently observed (2). AITD may result in hyperthyroidism, subclinical dysfunction or, most commonly, in hypothyroidism. The common hypothyroidism signs and symptoms include deterioration of well-being, excessive weight gain, dry skin, hair loss and many more. Moreover, AITDs may be a prelude to the subsequent development of thyroid hormone disorders. There is no ideal test for diagnosis of AITDs. Currently the diagnosis of AITD is based on the coexistence of clinical symptoms, presence of antibodies against thyroid antigens (thyroperoxidase, thyroglobulin) and characteristic ultrasound image (3). Therefore, it is undoubtedly worth looking for new methods of diagnosing and monitoring the disease. AITD can be associated with other autoimmune diseases in the same patient such as vitiligo, chronic autoimmune gastritis, rheumatoid arthritis or polymyalgia rheumatica (4).

It is a well-known fact that positron emission tomography (PET) is an effective tool in the assessment of thyroid focal lesions (5), especially in the diagnosis of advanced differentiated thyroid carcinoma (6).

However, we also know that the PET scan may be useful in the imaging of inflammatory lesions including thyroiditis (in some of the previous studies this case has been referred as “false-positive PET uptake”) (7, 8). Under normal conditions, uptake of FDG in the thyroid tissue is low or absent. The PET scans may present increased focal or diffuse pattern of FDG uptake. Diffusely increased uptake of FDG may be associated with AITDs or with the hypothyroidism (9–12). Focal increased uptake of FDG represents higher risk of malignancy (13).

PET scan is not a standard test used in diagnosing and monitoring of AITD patients. However, results of this imaging studies may be valuable in some cases.

The aim of this study was to assess if PET scan may be useful for the assessment of the thyroid gland in patients with AITD.

## Materials and Methods

We conducted 18-FDG-PET scan in twenty patients of our endocrinology clinic between September and November 2018, in which we diagnosed Hashimoto's in advance. The disease has been identified by elevated thyroid peroxidase antibodies level and by the hypoechoic pattern of the thyroid gland assessed by ultrasound imaging. Thyroid ultrasonography was performed using the Sonoscape E2 ultrasonograph device with 7.5 MHz linear probe.

Control subjects were six patients (exclusively women) who have been in carcinoma remission and underwent neck imaging with a PET scan due to reasons other than assessment of thyroid disease. AITDs had been excluded in all control group subjects. All control group subjects had their thyroid glands intact, none of them had a medical history of thyroid disorders.

Written informed consent was obtained from the every participant of this study. Bioethics Committee of the Nicolaus Copernicus University in Torun functioning at Collegium Medicum in Bydgoszcz stated their positive opinion on our research.

To assess the ^18^FDG uptake, the SUV-max was measured in the thyroid area. Nuclear medicine imaging was performed on the whole-body high-resolution PET/CT scanner Biograph 6. The images were acquired 60 min after radiotracer administration. To ensure the results proper interpretation, nuclear medicine specialist assessed them.

All data obtained were subjected to a statistical analysis with usage of STATISTICA 13.1. Differences in SUV-max values between a group of AITDs subjects and a group of healthy subjects were analyzed by the Mann-Whitney *U*-test and the aforementioned difference has been considered statistically significant at the *p*-value <0.05.

## Results

**Figure 1:**
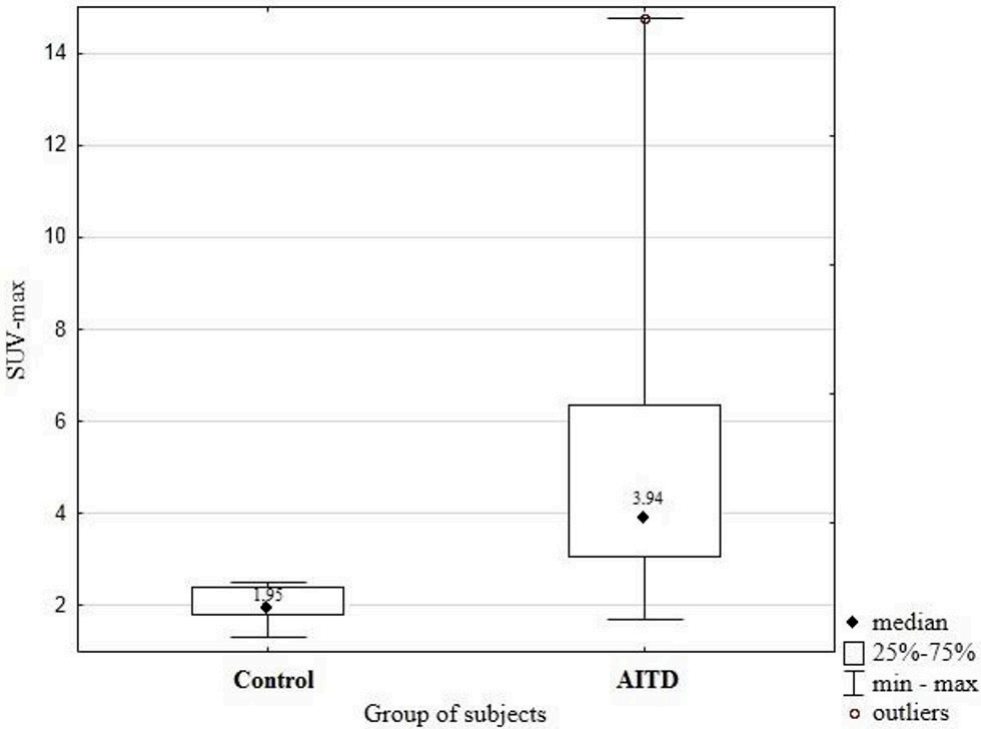
Difference of SUV-max between group of patients with AITD and control group subjects.

Median age of subjects with Hashimoto's thyroiditis was 41.56 ± 13.09 years. There were no age-related statistical differences in the ^18^FDG uptake in thyroid parenchyma (*p* = 0.57). The SUVmax has been measured in the thyroid area for all the subjects included in the study. SUV-max was significantly higher in patients with an AITD than in control subjects (median SUV-max 3.94 vs. 1.95; *p* = 0.005). The differences between SUV-max in the group of patients with an AITD- Hashimoto's thyroiditis and in control group patients was presented in Figure 1.

## Discussion

Positron emission tomography (PET) scan is widely used and a valuable method in work-up of patients with thyroid carcinoma or cancer screening in healthy patients (14). Nowadays, there are more than 5000 PET-CT systems available all over the world. In clinical oncology PET-CT may be used as a diagnostic tool which may reduce amount of unnecessary surgical interventions (15).

PET-CT scanning uses isotopes such as oxygen-15, fluorine-18 or gallium-68. In our research we used 18-fluorodeoxyglucose (^18^F-FDG) as a radiotracer. ^18^F-FDG has short half-live (~110 min) and it has been established that increased uptake of ^18^F-FDG was noted in cells with enhanced metabolism such as inflammatory cells and cancer cells measured as the maximum standardized uptake value (SUVmax) (10, 16, 17).

Most studies about thyroid PET scan have been focused on differentiation between benign and malignant lesions such as: thyroid incydentalomas or thyroid papillary carcinoma (17–19). Nevertheless, there have been only few reports of autoimmune thyroid disorders (AITDs) ^18^F-FDG -PET scan. Akosmana and al. described thyroid lymphoma associated with Hashimoto's disease in 44-year-old man. PET scan performed in this patient showed increased diffuse FDG uptake in thyroid gland (SUVmax−17.6). After treatment—third cycle of chemotherapy—FDG uptake by thyroid remained increased (SUVmax−5.1) (20). While Schmid et al. reported increased 18F-FDG uptake mimicking thyroid cancer in a patient with Hashimoto's thyroiditis (21).

The most common of AITDs in Poland is Hashimoto's thyroiditis, its prevalence has been estimated at 2%. It is frequent to notice hormonal abnormalities as hypothyroidism or presence of elevated thyroid antibodies serum level before the occurrence of the clinical symptoms such as weight gain, hair loss, mental disorders (3). In our research we studied if ^18^F-FDG PET scan can differentiate normal thyroid parenchyma from parenchymal changes of the thyroid gland in patients with Hashimoto's thyroiditis.

We evaluated 20 patients (17 women, 3 men) with Hashimoto's thyroiditis which has been diagnosed by the presence of elevated thyroid antibodies serum level and thyroid ultrasound imaging. Among all included subjects, 12 patients presented a normal TSH serum level and 8 patients presented an elevated TSH concentration. All subjects presented a normal triiodothyronine and thyroxine serum levels. None of the subjects were treated with levothyroxine before the ^18^F-FDG PET scan. Control subjects consisted of patients who have been in carcinoma remission and simultaneously underwent neck imaging with a PET scan due to reasons other than diagnosing or evaluating thyroid diseases.

The SUVmax of the thyroid parenchyma was measured using ^18^F-FDG PET. The SUVmax was significantly higher in subjects with AITD than in control subjects (4.25 (IQR 2.79–5.91 vs. 1.76 (IQR 1.33–2.36), *p* = 0.05). There was no significant SUVmax differences between patients who had elevated TSH concentration and normal TSH serum level. The highest reported SUVmax (14, 76) was presented by a patient with no hormonal abnormalities and slight clinical manifestations of the thyroid disorder. Each of 20 subjects presented diffuse type 18 -FDG uptake.

Yasuda et al. (8) and consecutively Seji et al. (22) have reported that diffuse type of thyroid 18-FDG uptake pattern may be associated with chronic thyroiditis. Yasuda et al. (8) also suggested that subclinical chronic thyroiditis may be diagnosed accidentally during whole-body PET scan. According to the results obtained and presented by Choi et al. (23) diffuse thyroid ^18^F-FDG uptake pattern most likely indicates benign thyroid lesion. Our results also indicate that diffuse ^18^F-FDG pattern uptake may support AITDs diagnosis.

The mechanism of ^18^F-FDG uptake in AITD is still unknown. In a research focused on activated inflammatory cells, an increased FDG uptake has been observed, probably due to enhanced expression of the Glucose Transporters Type 1 (GLUT-1), described by Chakrabarti and al. in human peripheral blood lymphocytes enriched in T cells after phytohemagglutinins stimulation (24).

The limitations of our study was a small number of performed PET scans due to low availability and high cost of PET imaging. In Poland there are 26 PET-CT systems per 37,4 milions citizens.

Summing it up, ^18^F-FDG -PET scan in AITDs patients shows abnormal diffuse FDG uptake pattern in thyroid parenchyma. Abnormal thyroid PET scan pattern may indicate autoimmune diseases in subjects with no previous medical history or may support the diagnosis of an AITD in patients with subtle clinical signs with normal hormone levels and/or elevated thyroid antibodies. Although, we judge it necessary to obtain more data about the usage of PET imaging in the management and follow-up of the AITDs. Moreover, it seems to be crucial to know the techniques to differentiate ^18^F-FDG -PET uptake patterns in patients with neoplastic lesions associated with AITD. The American Thyroid Association (ATA) recommends that nodules which has at least 10 mm in size should be investigated with ultrasound (US) and needle aspiration (FNAB) (13).

## Author Contributions

BM and RJ concept and design. ZS ultrasound imaging. SS, RG, RJ, and RŁ recrutation of patients. SS, RG, and RJ analysis and interpretation of data. SS, RG, and RJ manuscript writing. RJ review of final manuscript. All authors literature review and refinement of manuscript.

### Conflict of Interest Statement

The authors declare that the research was conducted in the absence of any commercial or financial relationships that could be construed as a potential conflict of interest.
